# Banxia-Yiyiren alleviates insomnia and anxiety by regulating the gut microbiota and metabolites of PCPA-induced insomnia model rats

**DOI:** 10.3389/fmicb.2024.1405566

**Published:** 2024-11-07

**Authors:** Liang Wang, Xiaorong Qi, Shuo Wang, Chujiao Tian, Tao Zou, Zihan Liu, Qi Chen, Yingfan Chen, Yunshan Zhao, Shaodan Li, Minghui Yang, Ningli Chai

**Affiliations:** ^1^Department of Gastroenterology, First Medical Center, Chinese PLA General Hospital, Beijing, China; ^2^The 955th Hospital of the Army, Qamdo, Tibet, China; ^3^Department of Chinese Medicine, Sixth Medical Center, Chinese PLA General Hospital, Beijing, China

**Keywords:** Banxia, Yiyiren, insomnia, anxiety, gut microbiota, metabolites, PCPA

## Abstract

**Objective:**

This study aims to clearly define the effects of Banxia-Yiyiren on the gut microbiota and its metabolites in a para-chlorophenylalanine-induced insomnia model and the possible underlying mechanisms involved.

**Materials and methods:**

We employed 16S ribosomal ribonucleic acid (rRNA) gene sequencing combined with metabonomic analysis to explore the mutual effects of the PCPA-induced insomnia model and the gut microbiota and the intrinsic regulatory mechanism of Banxia-Yiyiren on the gut microbiota and metabolites in the PCPA-induced insomnia model.

**Results:**

Banxia-Yiyiren was identified by mass spectrometry to include amino acids, small peptides, nucleotides, organic acids, flavonoids, fatty acids, lipids, and other main compound components. The elevated plus maze (EPM) test results revealed that high-dose Banxia-Yiyiren may increase willingness to explore by improving anxiety-like symptoms caused by insomnia. Through 16S rRNA gene sequencing, at the phylum level, compared with those in G1, the relative abundances of *Bacteroidota* and *Proteobacteria* in G2 increased, whereas the relative abundance of *Firmicutes* decreased. At the genus level, compared with those in G1, the relative abundances of *Prevotella_9*, *Prevotella*, *Ralstonia*, *Escherichia-Shigella*, and *UCG-005* in G2 increased, whereas the relative abundances of *Lactobacillus*, *Ligilactobacillus*, *Alloprevotella*, *Blautia*, and *Prevotellaceae_NK3B31_group* decreased. The metabolomics analysis results revealed 1,574 metabolites, 36.48% of which were classified as lipids and lipid-like molecules, 20.76% as organic acids and their derivatives, and 13.36% as organic heterocyclic compounds. The correlation between the top 20 differentially abundant metabolites in the G1–G2 groups was greater than that between the G3–G2 and G6–G2 groups. Kyoto Encyclopedia of Genes and Genomes (KEGG) enrichment analysis revealed that the main differentially abundant metabolites in each group were significantly enriched in various pathways, such as amino acid metabolism, adenosine triphosphate (ATP)-binding cassette (ABC) transporters, protein digestion, and absorption. Additionally, there was a significant Pearson correlation between the genus-level differences in the gut microbiota and the differentially abundant metabolites among the G1–G2, G3–G2, and G6–G2 groups.

**Conclusion:**

This study preliminarily verified that the PCPA-induced insomnia model is closely related to gut microbial metabolism and microecological disorders, and for the first time, we confirmed that Banxia-Yiyiren can act on the gut microbiota of PCPA-induced insomnia model rats and alleviate insomnia and anxiety by regulating the species, structure, abundance, and metabolites of the gut microbiota.

## 1 Introduction

Insomnia is a common sleep disorder that includes the following clinical features: difficulty falling asleep repeatedly, insufficient sleep, difficulty maintaining sleep after waking up early or waking up, susceptibility to fatigue, and unsatisfactory sleep quality (Li X. et al., 2023). The prevalence of insomnia is generally high, and serious cases may lead to cardiovascular disease, diabetes, depression, cognitive dysfunction, and other diseases; as such, insomnia has become a global public health problem. At present, the common pharmacological treatments for insomnia are far from satisfactory for clinical application because of their various adverse effects (Wang et al., 2023). Therefore, developing potential, effective, safe drugs from Chinese natural herbs with few side effects is highly important for the clinical treatment of insomnia (Liu et al., 2022).

Traditional Chinese medicine (TCM) has a definite therapeutic effect and a low incidence of adverse reactions, making it an effective intervention for treating insomnia (Wu et al., 2022). The Chinese herbal medicine pair Banxia-Yiyiren originates from the Banxia–Shumi decoction (Huangdi Neijing) and is known as the “ancestral formula for treating insomnia.” In clinical practice, Banxia-Yiyiren is commonly mixed at a ratio of 1:2 and consists of *Pinellia ternata* and Coix seeds. For approximately 2000 years, Banxia–Shumi decoction has been widely used to treat insomnia, and its specific forms include its use alone or in combination with other traditional Chinese and Western medicines, which have good therapeutic effects (Lin et al., 2021).

The interaction between the gut microbiota and sleep has a bidirectional regulatory effect (Zhou et al., 2022). On the one hand, the gut microbiota metabolizes various neurotransmitters, cytokines, and metabolites, such as 5-HT, GABA, melatonin, and other sleep-related compounds. On the other hand, insufficient sleep or sleep deprivation can affect the gut microbiota composition and metabolic function, leading to microbial imbalance (Wang X. et al., 2024). With the advancements in sequencing and metabolomics technology, the application of these materials in the study of medicinal and edible homologous Chinese medicines is increasing.

**Figure 1:**
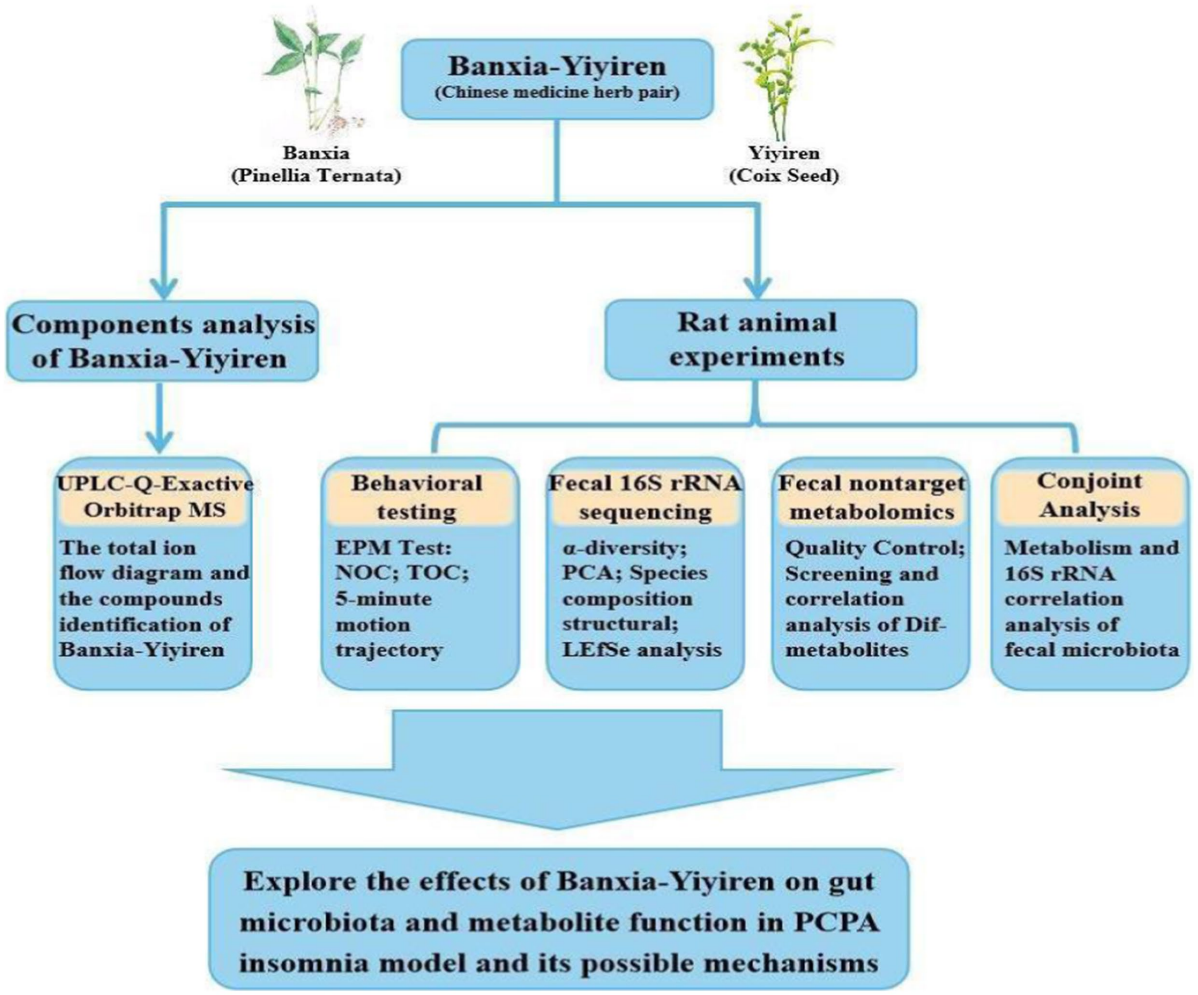
Overall flowchart of this study.

At present, the gut microbiota and metabolic mechanism related to Banxia-Yiyiren, a Chinese herbal medicine with dual medicinal and edible characteristics, are not yet clear, and research on its sleep regulatory mechanism is relatively limited. Therefore, based on the above research and the PCPA-induced insomnia model, this study integrated ultraperformance liquid chromatography (UPLC)-Q Exactive Orbitrap MS, 16S rRNA, and metabolomics to explore the regulatory effect of Banxia-Yiyiren on the gut microbiota and its metabolites in insomnia rats to reveal the mechanism of its anti-insomnia effect (Figure 1).

## 2 Materials and methods

### 2.1 Drugs, reagents, and preparation

#### 2.1.1 Banxia-Yiyiren

Provided and identified by the TCM Department, Chinese PLA General Hospital. The materials were prepared by mixing 10 g of Banxia (Anhui Puren Pharmaceutical Co., Ltd., batch number 2107158, origin: Gansu) and 20 g of Yiyiren (Beijing Lvye Pharmaceutical Co., Ltd., batch number 20082401, origin: Hebei). Detailed selection, preparation methods, and decoction procedure can be found in Appendix 1.

Following the TCM methods, the materials were decocted according to the equivalent dose ratio of human and rat body surface area; the products were concentrated to concentrations of 0.16 g/mL (low dose), 0.32 g/mL (medium dose), and 0.64 g/mL (high dose) of the raw drug and stored in a refrigerator at 4°C for future use. Diazepam (Beijing Yimin Pharmaceutical Co., Ltd., batch number H11020898) was used as a positive control drug, with a hypnotic dose of 0.5 mg/kg/day. The equivalent dose was calculated from the surface area between the human body and the rats, and the concentration of diazepam was 0.83 mg/kg. The samples were stored in a refrigerator at 4°C for future use.

#### 2.1.2 PCPA

4-chloro-DL-phenylalanine (purchased from Sigma Corporation in the United States; specification 5 g; product batch number 1003164450) was dissolved in weakly alkaline saline water (pH 7–8). The samples were stored in a refrigerator at 4°C for future use.

### 2.2 UPLC-Q Exactive Orbitrap MS

#### 2.2.1 Chromatographic methods

We used an ultrahigh-performance liquid-phase UHPLC (Vanquish Flex, Thermo Fisher Scientific) system. A Thermo Scientific™ Q Active instrument was used for quadrupole electrostatic field orbital trap high-resolution mass spectrometry, and the ± electrospray ionization (ESI) detection mode was used for chromatographic mass spectrometry analysis of Banxia–Yiyiren. The specific parameters were set according to the relevant literature. Mass spectrometry conditions: Using XCalibur 4.3 and Compound Discoverer 3.2 chemical workstations from Thermo Fisher in the United States for data correlation processing, a total ion map of Banxia-Yiyiren was obtained. Compared with existing databases, preliminary predictions were made for each chromatographic peak in the above results.

### 2.3 Experimental animals and the PCPA-induced insomnia model

#### 2.3.1 Experimental animals

This animal experimental study was approved by the Experimental Animal Ethics Committee of the Chinese PLA General Hospital (ethical code: 2020-X16-102). Thirty-six male SPF-grade Wistar rats were purchased from Sibeifu (Beijing) Biotechnology Co., Ltd. (animal qualification certificate number: SCXK (Beijing) 2019-0010), weighed 160 ± 10 g, identified and raised by the Medical Experimental Animal Center of Chinese PLA General Hospital. The animals were adaptively fed for 1 week in an animal feeding room (with an ambient temperature of 25 ± 2°C, relative humidity of 50 ± 5%, and a 12/12-h light/dark cycle). Throughout the experiment, the animals’ feeding process was made to strictly adhere to relevant national guidelines for animal management and protection.

#### 2.3.2 PCPA-induced insomnia model

According to the literature and previous studies by the research team, a PCPA-induced insomnia rat model was used. PCPA (300 mg/kg) was injected intraperitoneally once a day for 3 days. The normal group was injected with the same amount of normal saline for consecutive 3 days. At 48 h after the first injection of PCPA, the circadian rhythm of the rats in the model group disappeared, and both day and night were constantly active. The insomnia model was evaluated in combination with the classical pentobarbital sodium correction test, and the rats in the model group were intraperitoneally injected with pentobarbital sodium (35 mg/kg) at 8–9 a.m. on the third day. The sleep latency period and duration from injection to reversal disappearance were recorded (sleep time ranged from reversal disappearance to the suitable time of 30 s when the rats could not maintain the supine position after turning over). If the model group was compared with the normal group, the difference was statistically significant, indicating the model’s success.

### 2.4 Experimental grouping and intervention

All the rats were randomly divided into 6 groups via a random number table: G1 (normal group), G2 (model group), G3 (diazepam group), G4 (low-dose Banxia-Yiyiren group), G5 (medium-dose Banxia-Yiyiren group), and G6 (high-dose Banxia-Yiyiren group). G1 and G2 were given the same volume of weakly alkaline 0.9% sodium chloride by gavage. The rats in each group were treated with 1 mL/100 g of the corresponding medication by gavage for consecutive 7 days.

### 2.5 Elevated plus maze (EPM) test

Six rats from each group were selected for this test 12 h after the end of the last rat gavage to evaluate the anxiety status of the insomnia model. Super Maze and Visu Track animal behavior analysis software were used to track the motion trajectory within 5 min. The following parameters were recorded: the number of times the rats entered the open arm entry (OE), the number of open arms (OT), the number of times they entered the closed arm entry (CE), and the length of the closed arm (CT). The percentage of open arm entry was calculated as OE% = OE/(OE + CE) × 100%, and the percentage of open arm time was calculated as OT% = OT/(OT + CT) × 100%.

### 2.6 Sample collection

After 7 days of intragastric administration and completion of rat behavioral testing, the gut feces of each group of rats were collected on a sterile operating platform (disinfected before each operation to avoid contamination), placed in a prelabeled 2 mL sterile frozen tube, immediately frozen in liquid nitrogen, and then transferred to a −80°C freezer for testing.

### 2.7 Analysis of the gut microbiota

#### 2.7.1 16S rRNA gene sequencing of the gut microbiota

Six rats were selected from each group for gut microbiota 16S r*RNA* analysis. According to the kit’s instructions (DP712) from TianGen Biotech (Beijing) Co., Ltd., DNA was extracted from rat feces, and the purity of the extracted DNA was checked via agarose gel electrophoresis. Then, PCR amplification was performed (using 5′-CCTAYGGGRBGCASCAG-3′ and 5′-GACATCNNGGGTTATCTAAT-3′) to complete the entire library preparation. Double-terminal sequencing was performed on the constructed library via the Illumina NovaSeq sequencing platform. Finally, species annotation and abundance analysis of the gut microbiota were conducted.

#### 2.7.2 Metabolomic analysis of the gut microbiota

Beijing Nuohe Zhiyuan Technology Co., Ltd. conducted the metabolomic analysis of the feces. Non-targeted metabolomics research is based on liquid chromatography–mass spectrometry (LC–MS) technology (Want et al., 2010; Dunn et al., 2011). The experimental process included sample metabolite extraction, LC–MS/MS detection/metabolite qualitative and quantitative analysis, and data analysis. First, the mass spectrometry conditions were strictly set according to the established standards, and the original files (raw) obtained from mass spectrometry detection were imported into Compound Discoverer 3.3 software for spectrum processing and database searches to obtain qualitative and quantitative results for the metabolites. Then, quality control is carried out on the data to ensure the accuracy and reliability of the results. Next, we conducted multivariate statistical analyses, such as principal component analysis (PCA) and partial least squares discriminant analysis (PLS-DA), on the metabolites to reveal the differences in metabolic patterns among the different populations. Moreover, we used hierarchical clustering analysis (HCA) and metabolite correlation analysis to reveal the relationships between the samples and metabolites. Finally, the KEGG database.^1^

The HMDB^2^ and LIPIDMaps databases^3^ annotate bioinformatics-related functions such as metabolic pathways associated with identified metabolites (Sellick et al., 2011).

#### 2.7.3 Correlation analysis of 16S rRNA and the metabolome

We conducted Pearson correlation analysis of the differential microbiota (16S rRNA analysis at the genus level) and differentially abundant metabolites (metabolomics). We plotted a correlation analysis heatmap between the differential microbiota (top 10) and differentially abundant metabolites (top 20). Finally, we selected results that satisfied |*ρ*| ≥ 0.8 and *p* < 0.05 while generating a scatter plot.

### 2.8 Statistical analysis

All the analyses were performed via Graph Pad Prism 7.0 software, SPSS (SPSS) 16.0 software, and the Novo Cloud platform.^4^ The data are presented as the means ± standard error of measurements (SEMs). The false discovery rate (FDR) error control method was used to correct *p*-values in the transcriptome and metabolome. Statistical differences were analyzed via one-way analysis of variance (ANOVA) and Student’s *t*-test. Differences with *p* < 0.05 were considered statistically significant.

## 3 Results

### 3.1 Banxia-Yiyiren as an ingredient

By the aforementioned chromatographic methods and mass spectrometry methods, UPLC-Q Exactive Orbitrap MS was used to qualitatively analyze the water extraction components of Banxia-Yiyiren under positive and negative ion detection modes, and a total ion flow diagram was generated (Appendix 2). Through accurate molecular weight calculations and ion fragmentation information, the main compound components, including amino acids, small peptides, nucleotides, organic acids, flavonoids, fatty acids, and lipids, were identified via mass spectrometry. Some compounds were confirmed by reference standards (Appendix 3).

### 3.2 EPM test

**Figure 2:**
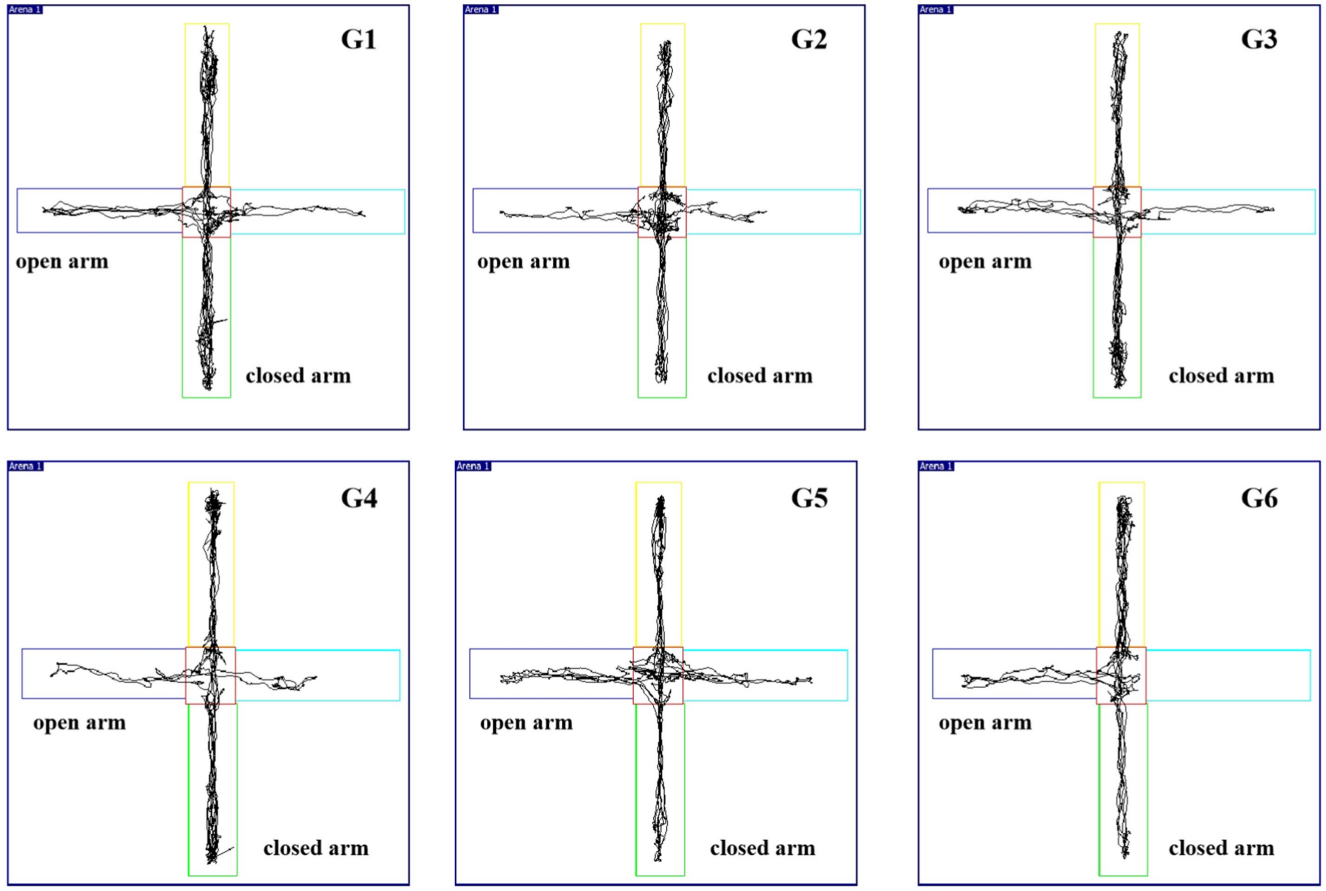
Trajectory of each group in the elevated plus maze (EPM) test.

Compared with those in G1, the percentages of OE and OT in G2 and G3 rats were significantly lower (*p* < 0.01). Compared with those of G2, the TOC contents of G3, G4, G5, and G6 were significantly greater (*p* < 0.05), as shown in Appendix 4 and Figure 2. These findings indicate a decrease in the exploratory ability of the model group. In general, the exploratory activities of the model group were significantly lower than those of the control group, and they had decreased cognitive function; both of these findings are considered insomnia-related behaviors.

### 3.3 Gut microbiota analysis

**Figure 3:**
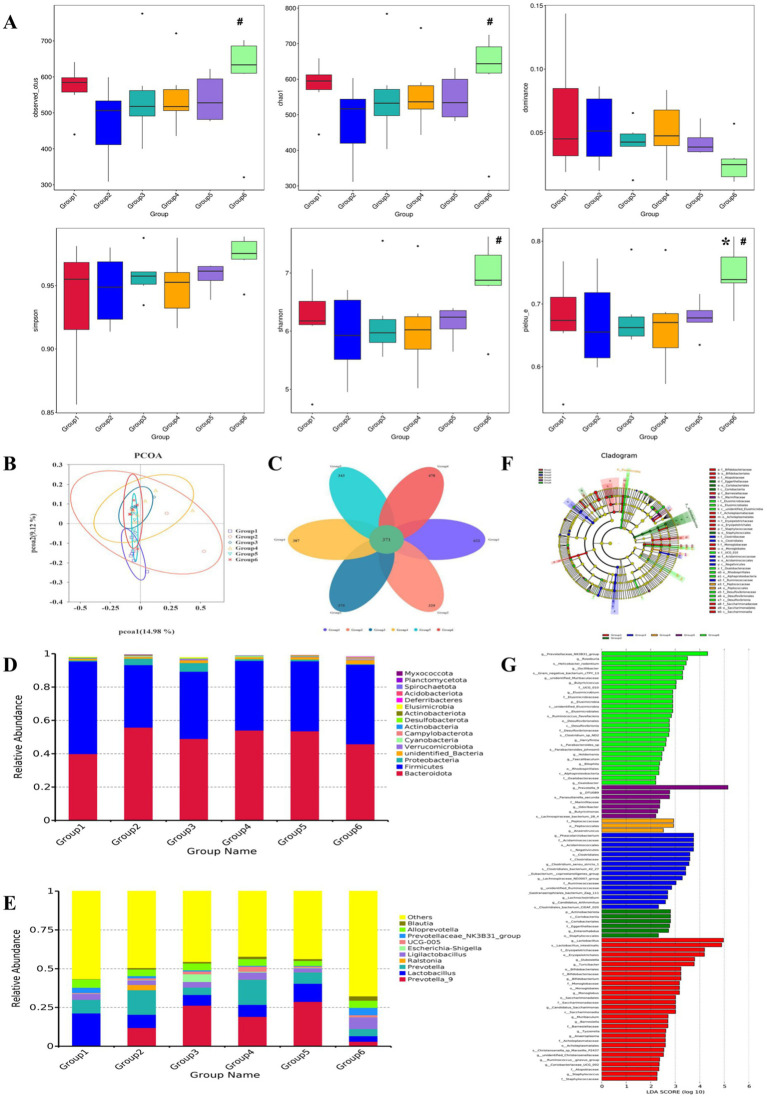
The α-diversity, principal coordinates analysis (PCoA), species composition structure analysis, and relative abundance of the gut microbiota. **(A)** α-Diversity of the gut microbiota (*p < 0.05 vs. G1; #p < 0.05 vs. G2); **(B)** unweighted UniFrac PCoA analysis chart of the gut microflora; **(C)** petals of the gut microflora; **(D)** relative abundance at the gut microflora phylum level; **(E)** relative abundance at the gut microflora genus level; **(F)** characteristic microbiota of each group. Notes: The large circles correspond to the hierarchical relationships of phyla, class, order, family, and genus from the inside out. The larger the diameter of the small circle is, the greater its relative abundance. Species with no significant differences are marked in yellow, while different groups indicate the different species biomarkers. The red nodes represent the microbial groups that play essential roles in the red group, and the green nodes represent the microbial groups that play vital roles in the green group. **(G)** LDA bar chart of the gut microflora. Note: The vertical axis represents the gut microbiota with significant differences between groups, whereas the horizontal axis represents the LDA score of each microbiota. The size of the LDA score is proportional to the impact of different species.

The richness and evenness of species within each community were evaluated via a diversity measure: Observed_Otus, Chao1, and dominance were used to calculate microbial abundance; Simpson and Shannon indices were used to reflect the diversity of the sample microbiota; and Pielou_E was used to calculate species evenness. The dominance and Simpson indices of G1 and G2 were significantly different (*p* < 0.05), whereas the overall differences in the other groups of samples were not statistically significant (*p* > 0.05), as shown in Figure 3A.

As shown in Figure 3B, based on unweighted UniFrac principal coordinates analysis (PCoA) analysis of differences in the gut microbial community structure, G1 and G2 were separated in the PCoA diagram. This indicates a significant change in species abundance between G1 and G2. However, after intervention with each group of drugs, the distribution of samples in G3, G4, G5, and G6 gradually tended toward G1. These findings indicate that G3, G4, G5, and G6 have varying degrees of influence on the gut microbiota of G2.

To study the species composition of each sample, a visualized petal map of the OTU distribution was constructed (Figure 3C). The species shared by G1, G2, G3, G4, G5, and G6 included 371 OTUs. Among them, G1 had 622 unique OTUs, G2 had 329 unique OTUs, G3 had 379 unique OTUs, G4 had 387 unique OTUs, G5 had 343 unique OTUs, and G6 had 478 unique OTUs.

16S rRNA gene sequencing confirmed the relative abundance of the species at the phylum level (Figure 3D). Analysis of the composition of the bacterial community structure in each sample revealed that the main phyla included *Bacteroidota*, *Firmicutes*, and *Proteobacteria*. Compared with those in G1, the relative abundance of *Bacteroidota* in G2 tended to increase, that of *Firmicutes* tended to decrease, and that of *Proteobacteria* tended to increase. G4, G5, and G6 reversed the changes in the abundance of the above dominant bacteria, with G6 having the most significant effect. At the genus level (Figure 3E), the dominant genera with abundances in the top 10 included *Prevotella_9*, *Lactobacillus*, *Prevotella*, *Ralstonia*, *Ligilactobacillus*, *Escherichia*, *Shigella*, *UCG-005*, *Prevotelaceae_NK3B31_Group*, *Alloprevotella*, and *Blautia*, compared with those in the G1 and G2 groups. The relative abundances of *Prevotella*, *Ralstonia*, *Escherichia*, *Shigella*, and *UCG-005* tended to increase, whereas those of the other genera tended to decrease. In G4, G5, and G6, the trend of changes in the above bacterial genera improved, with the effects occurring more significantly in G5 and G6.

LEfSe analysis was used to compare and analyze the biomarkers with significant differences at different classification levels in each group, with a linear discriminant analysis (LDA) score set to 2. As shown in Figures 3F,G, a total of 91 differential species were identified, including 30 species in G1, 6 species in G2, 16 species in G3, 3 species in G4, 7 species in G5, and 28 species in G6. The characteristic microbiota with significant differences in G1 were *g_Lactobacillus*, *s_Lactobacillus_gutis*, *o_Erysipelotrichales*, *f_Erysipelotrichaceae*, *g_Turiciactor*, *g_Dubosella, f_Bifidobacteriaceae*, etc. The characteristic microbiota with significant differences in G2 were *o_Coriobacteriales*, *c_Coriobasteria*, *p_Actinobacteriota, f_Eggerthellaceae*, *g_Enterorhabdus*, and *o_Staphyloccales*. The characteristic microbiota with significant differences in G3 were *c_Negativities*, *o_Acidaminoscales*, *f_Acidaminococcaceae*, *g_Phascolarctobacterium*, *f_Clostridiaceae*, *o_Clostridiales*, and *g_Clostridium_Sensu_Stricto_Class 1*. The characteristic microbiota with significant differences in G4 were *o_Peptoccales*, *f_Peptococcaceae*, and *g_Anaerotruncatus*. The characteristic microbiota with significant differences in abundance in G5 were *g_Prevotella_9*, *S_Parasutterella_Secunda*, *f_Marinifilaceae*, *g_Butyrisimonas*, *s_Lachnospiraceae_Bacterium_28_4*, *g_Odoribacter, g_DTU089*, etc. The characteristic microbiota with significant differences in G6 were *g_Prevotelaceae_NK3B31_Group*, *g_Roseburia, g_Oscillibacter*, *s_Helicobacter_Rodentium*, *g_Unidentified_Muribaculaceae*, *s_Gram_Negative_Bacterium_CTPY_13*, *f_UCG_010, g_Butyricicoccus*, *f_Elusimicrobiaceae*, *g_Elusimicrobium*, *p_Elusimicrobia*, etc.

### 3.4 Metabolomic analysis

#### 3.4.1 Quality control (QC)

**Figure 4:**
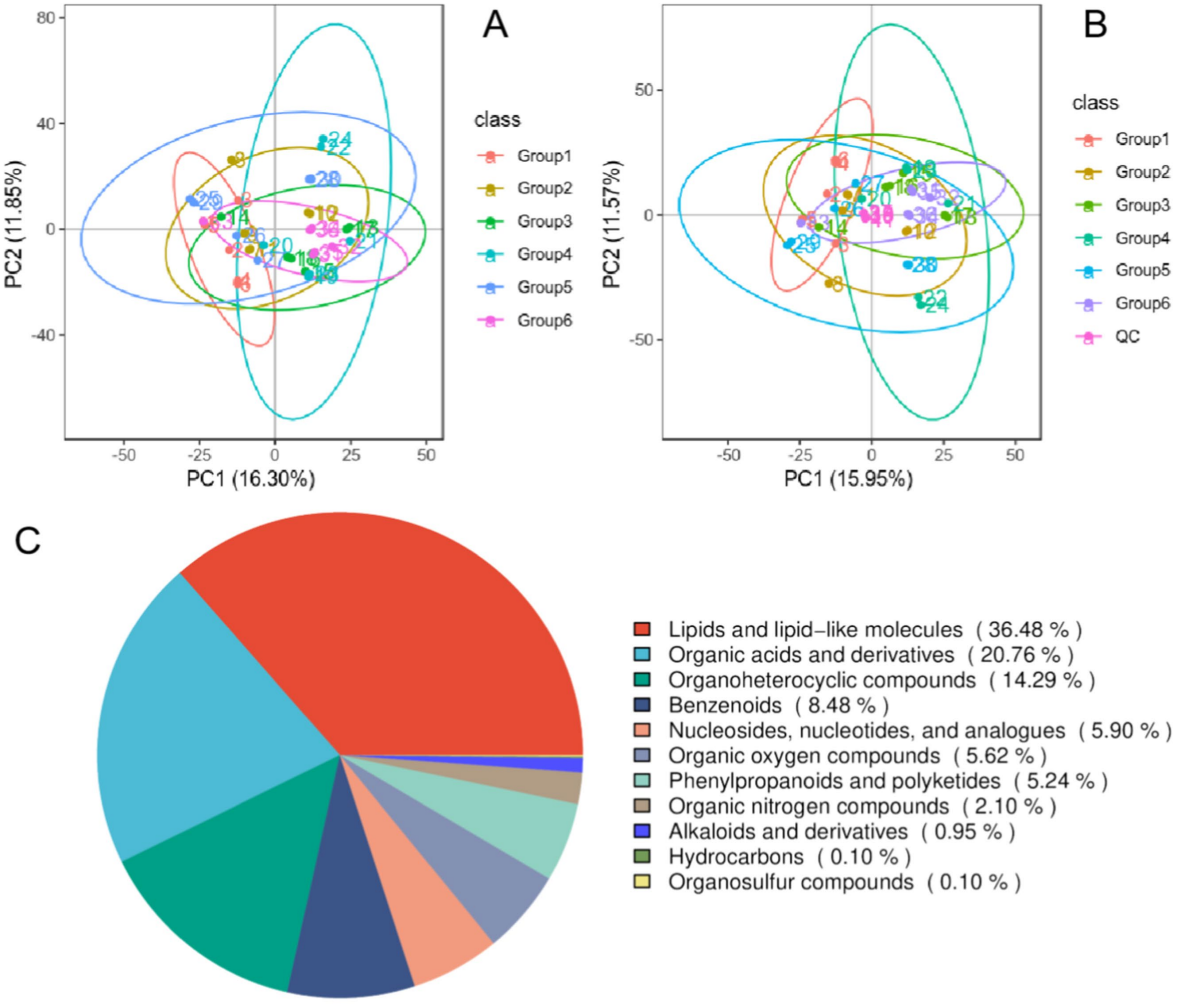
Quality control (QC) sample correlation analysis and total sample principal component analysis (PCA). **(A)** PCA score map for positive ion flow; **(B)** PCA score map for negative ion flow; **(C)** metabolite classification pie chart.

The PCA modeling method was used to detect the aggregation degree of the QC samples and subsequently evaluate the quality of the experimental data. According to the PCA of each group and QC sample, the distribution of QC samples in positive (negative) ion mode was dense, and the difference was small, indicating that the data quality of this experiment was reliable and that its stability and reproducibility were good (Figures 4A,B). In addition, PCA revealed no significant separation between the groups, indicating that the positive control drug and Banxia-Yiyiren did not significantly affect the changes in the gut-fecal metabolism spectrum. A custom-built standard product database was used to search for and match the detailed information of metabolites in the samples. A total of 1,574 metabolites were identified (1,065 in ESI+ mode and 509 in ESI− mode). According to the chemical taxonomic information, 36.48% of the metabolites were classified as lipids or lipid-like molecules, 20.76% were organic acids or derivatives, and 13.36% were organic heterocyclic compounds (Figure 4C).

#### 3.4.2 Screening for differentially abundant metabolites

**Figure 5:**
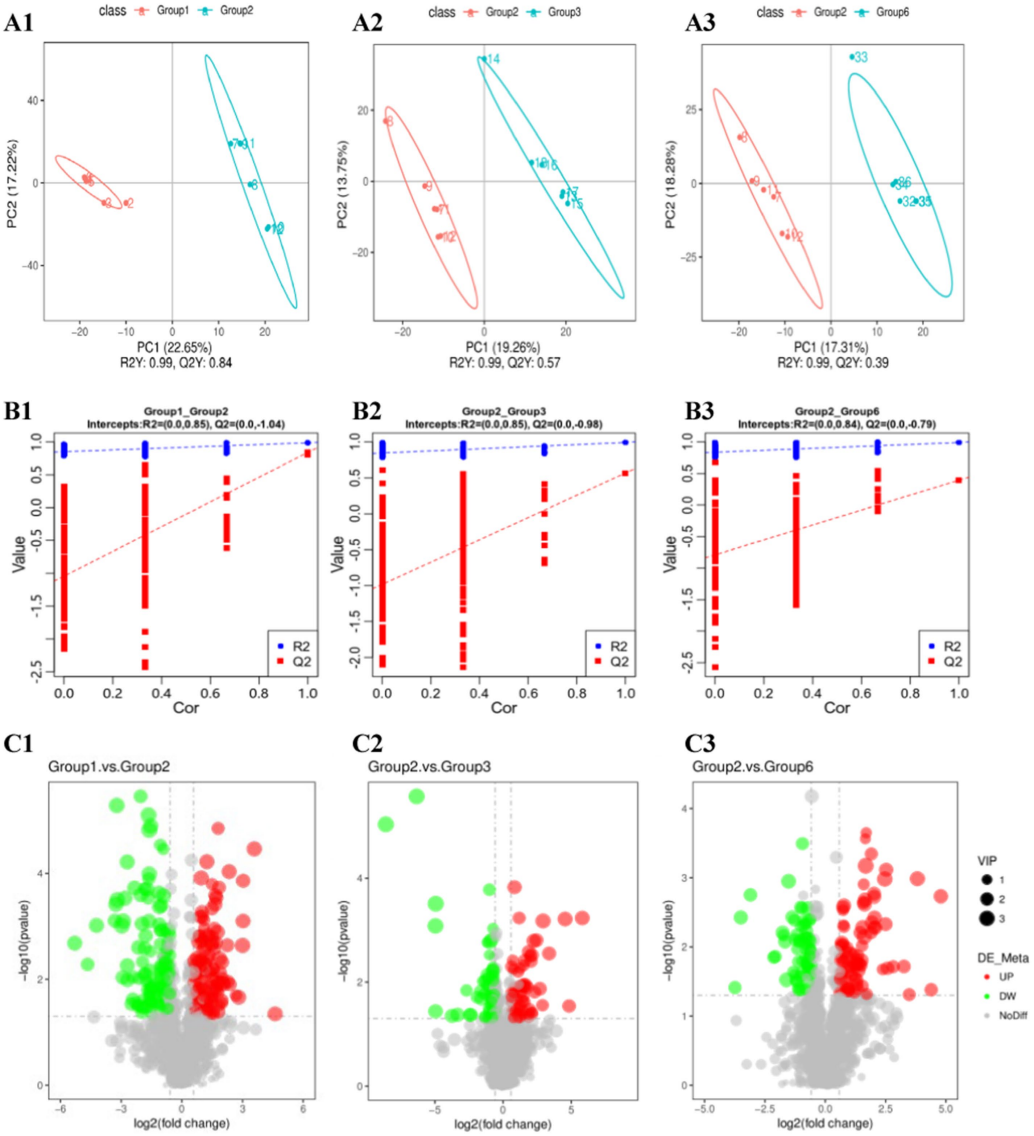
Metabolomic analysis of cecum contents. **(A1–A3)** PLS-DA; **(B1–B3)** alignment test plots of the OPLS-DA model; **(C1–C3)** volcano plots of the differentially abundant metabolites.

To explore the differentially abundant metabolites between groups, we used PCA and partial least squares discriminant analysis (PLS-DA) models to analyze, observe, and verify the stability of the model. PCA and PLS-DA revealed a clear separation trend between G1–G2 and G6–G2, whereas there was no separation trend between G3–G2 (Figures 5A1–A3).

By determining the criteria for differentially abundant metabolites (FC > 1.5 and *p* < 0.05) and plotting volcano plots (Figures 5B1–B3), we found that there were significantly more differentially abundant metabolites between G1–G2 than between G3–G2, and G6–G2. We performed logarithmic conversion on the fold change values of different metabolites in each group. We selected the top 20 metabolites that were upregulated and downregulated between each group for the matchstick plot display. There were mainly upregulated cyclic and succinic acids between the G1 and G2 groups, whereas L-lysine and 8,8′-dicarboxy-1,1′-binaphthalene were downregulated. In G3 and G2, the expression of rhamnetin and 4-hydroxynordiazepam was upregulated, that of succinic acid and erythrono-1,4-lactone was downregulated, that of H-Trp-NH2 and stearic acid was upregulated, and that of succinic acid and erythrono-1,4-lactone was downregulated (Figures 5C1–C3; Table 1).

**Table 1:** Important differentially abundant metabolites among major groups (top 10).

Group	Name of differentially abundant metabolites	Formula	Molecular weight	Retention time (min)	m/z	*p*-value	Up or down
G1 vs. G2	FAHFA 15:0/17:2	C_32_ H_58_ O_4_	506.43054	9.655	505.42326	3.50E-06	Down
	FAHFA 14:0/16:2	C_30_ H_54_ O_4_	478.39916	9.2	477.39188	5.21E-06	Down
	Tetramethylpyrazine	C_8_ H_12_ N_2_	136.10041	2.522	137.10768	7.92E-06	Down
	Lauric acid ethyl ester	C_14_ H_28_ O_2_	228.20822	9.193	227.20094	1.26E-05	Down
	(3*R*)-8-Hydroxy-3-(4-methoxyphenyl)-3,4-dihydro-1*H*-2-benzopyran-1-one	C_16_ H_14_ O_4_	135.04332	5.54	134.036	1.41E-05	Up
	2-Isobutyl-3-methoxypyrazine	C_9_ H_14_ N_2_ O	166.11106	2.648	167.11834	1.51E-05	Down
	Nicotinamide *N*-oxide	C_6_ H_6_ N_2_ O_2_	138.04325	2.166	139.05052	2.91E-05	Down
	8,8′-Dicarboxy-1,1′-binaphthalene	C_22_ H_14_ O_4_	342.09188	4.955	341.08461	3.45E-05	Up
	γ-Aminobutyric acid (GABA)	C_4_ H_9_ N O_2_	103.06412	1.298	104.0714	3.47E-05	Down
	Citraconic acid	C_5_ H_6_ O_4_	130.02529	1.822	129.01798	6.02E-05	Up
G2 vs. G3	4-Hydroxynordiazepam	C_15_ H_11_ Cl N_2_ O_2_	286.05164	5.737	287.05892	2.66E-06	Down
	Rhamnetin	C_16_ H_12_ O_7_	316.06209	5.906	317.06936	9.17E-06	Down
	Lipoamide	C_8_ H_15_ N O S_2_	205.05924	2.046	188.05594	1.49E-04	Up
	2-(2-Carboxy-2-methylpropyl)-4,6-dimethylbenzoic acid	C_14_ H_18_ O_4_	250.11988	5.917	249.11259	1.66E-04	Down
	2-(3,4-Dimethoxyphenyl)quinoline	C_17_ H_15_ NO_2_	303.06749	5.892	304.07476	3.09E-04	Down
	3-(3-nitrophenyl)-2-phenylacrylic acid	C_15_ H_11_ N O_4_	290.04567	5.37	289.03836	5.80E-04	Up
	Succinic acid	C_4_ H_6_ O_4_	118.02511	2.486	117.01783	5.80E-04	Up
	3-(3-Bromo-4-hydroxy-5-methoxyphenyl)-2-cyanoacrylamide	C_11_ H_9_ Br N_2_ O_3_	295.98179	2.514	294.97451	6.08E-04	Up
	*N*-{6-[(5-Chloro-3-pyridyl)oxy]-3-pyridyl}-*N*′-methylurea	C_12_ H_11_ Cl N_4_ O_2_	278.05917	2.519	279.06644	6.62E-04	Up
	Morin	C_15_ H_10_ O_7_	302.04676	5.852	303.05403	8.26E-04	Down
G2 vs. G6	Phenethylamine	C_8_ H_11_ N	121.08964	5.048	122.09684	2.23E-04	Up
	Styrene	C_8_ H_8_	104.0631	5.048	105.0704	2.74E-04	Up
	Phosphoethanolamine	C_2_ H_8_ N O_4_ P	141.01951	1.266	142.02679	3.21E-04	Down
	GNK	C_12_ H_23_ N_5_ O_5_	299.16023	4.309	300.16751	4.54E-04	Up
	3-[3-(Methylthio)anilino]-1,3-dihydroisobenzofuran-1-one	C_15_ H_13_ N O_2_ S	271.06895	4.681	270.06167	6.72E-04	Up
	Citric acid	C_6_ H_8_ O_7_	192.02583	1.968	191.01855	7.65E-04	Up
	3-(3-Bromo-4-hydroxy-5-methoxyphenyl)-2-cyanoacrylamide	C_11_ H_9_ Br N_2_ O_3_	295.98179	2.514	294.97451	1.03E-03	Up
	*N*-{6-[(5-Chloro-3-pyridyl)oxy]-3-pyridyl}-*N*′-methylurea	C_12_ H_11_ Cl N_4_ O_2_	278.05917	2.519	279.06644	1.04E-03	Up
	2-Amino-1,3,4-octadecanetriol	C_18_ H_39_ N O_3_	317.29348	6.203	318.30075	1.13E-03	Down
	Acetophenone	C_8_ H_8_ O	120.05788	2.388	121.06516	1.25E-03	Up

FAHFA, fatty acid esters of hydroxy fatty acids.

To avoid overfitting the model, the grouping labels of each sample were randomly shuffled before modeling and prediction, and the model evaluation parameters (R2, Q2) were obtained through 7-fold cross-validation. The R2 values for G1–G2, G3–G2, and G6–G2 are greater than the Q2 values, and the intercept between the Q2 regression line and the *Y*-axis is less than 0. This finding indicates that the PLS-DA model does not have “overfitting” and can better describe the sample, which can be used to search for model biomarker groups (Appendix 5).

#### 3.4.3 Analysis of differentially abundant metabolites and correlation analysis of the gut microbiota and metabolites

**Figure 6:**
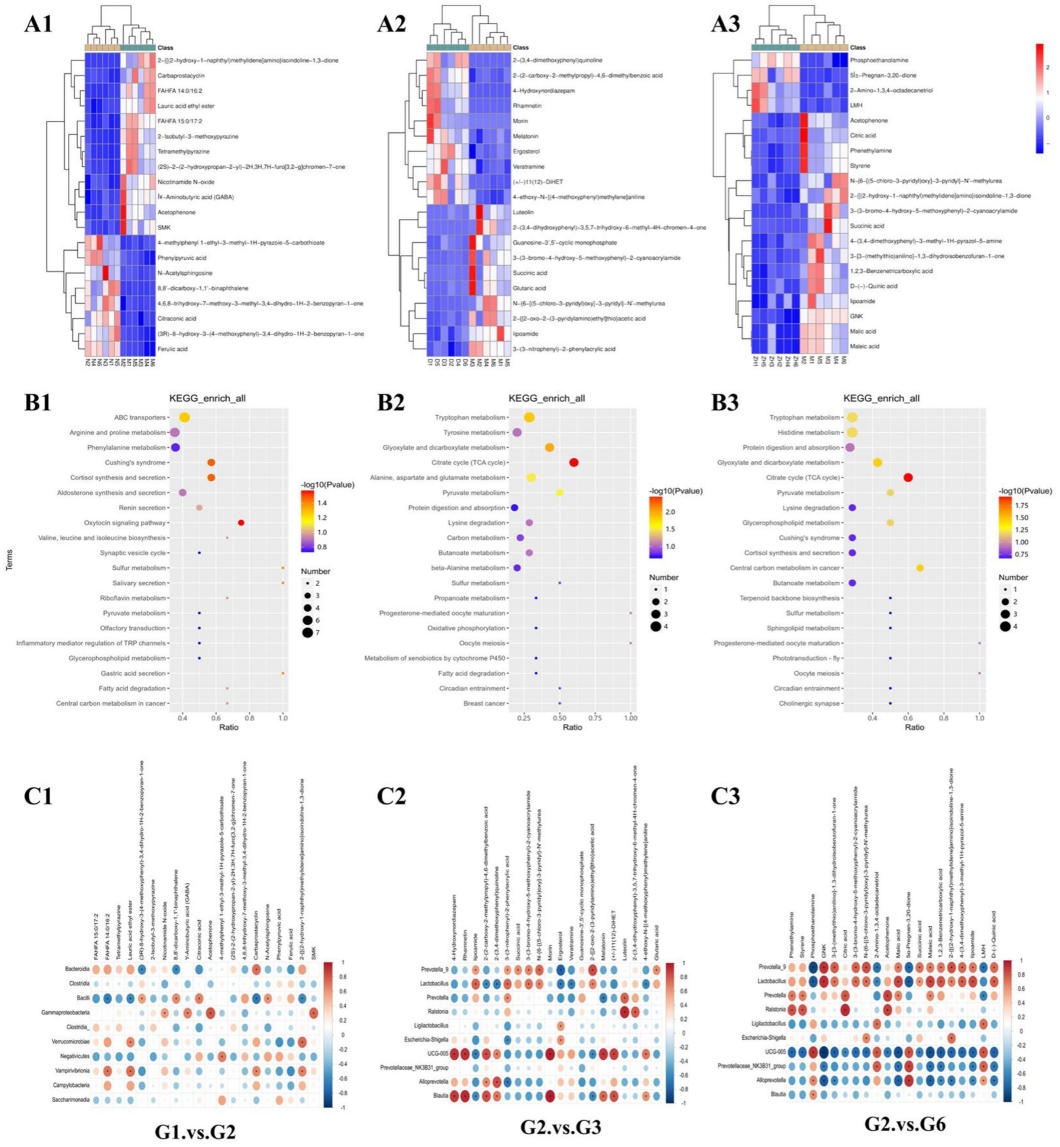
Heatmaps and enrichment pathways of important differentially abundant metabolites. **(A1–A3)** Heatmaps of important differentially abundant metabolites among major groups (top 20); **(B1–B3)** Kyoto Encyclopedia of Genes and Genomes (KEGG) enrichment pathway bubble chart of important differentially abundant metabolites among the major groups (top 20); **(C1–C3)** Heatmap of correlation analysis between significantly different microbial communities and significantly different metabolites at the genus level in each group (*p < 0.05).

A heatmap of the main differentially abundant metabolites between the groups is shown in Figures 6A1–A3. Compared with those in G1, ferulic acid, phenolpyruvic acid, and citronic acid in G2 decreased, whereas the contents of GABA, nicotinamide *N*-oxide, and lauric acid ethyl ester increased. Compared with those in G2, the contents of lipoamide, 3-(3-nitrophenyl)-2-phenylacrylic acid and 2-{[2-oxo-2-(3-pyridylamino)ethyl]thio}acetic acid in G3 decreased, whereas the contents of 2-(3,4-dimethoxyphenyl)quinoline, 4-hydroxynordiazepam, melatonin, and ergosterol increased. Compared with those in G2, the contents of GNK, malic acid, and maleic acid in G6 decreased, whereas the contents of phosphoethanolamine, 5β-Pregnan-3,20-dione, and 2-amino-1,3,4-octadecanetriol increased.

KEGG enrichment analysis of the top 20 differentially abundant metabolites in each group revealed that each group’s main differentially abundant metabolites were significantly enriched in various amino acid metabolism, ABC transporter, protein digestion, and absorption pathways (Figures 6B1–B3). Among them, the enriched KEGG pathways in G1–G2 were ABC transporters, arginine and profile metabolism, and phenoline metabolism. The enriched KEGG pathways in G2–G3 were related mainly to *Toxoplasma gondii* metabolism, tyrosine metabolism, glyoxylate metabolism, and dicarboxylate metabolism. The enriched KEGG pathways in G2–G6 were primarily related to *T. gondii* metabolism, histidine metabolism, protein digestion, and absorption.

The gut microbiotas of G1–G2, G3–G2, and G6–G2 were significantly correlated with differentially abundant metabolites. From G1–G2, we found that the PCPA-induced insomnia model was related mainly to the correlation between the 5 gut microbiota constituents and the 13 metabolites (Figure 6C1). After screening for |*ρ*| ≥ 0.8 and *p* < 0.05, we found that the major gut microbiota (*Blautia*, *UCG-005*, *Alloprevotella*, *Prevotella_9*, and *Ralstonia*) and the major metabolites (rhamnetin, 4-hydroxynordiazepam, morin, melatonin, 2-(3,4-dimethoxyphenyl)quinoline, luteolin, 2-{[2-oxo-2-(3-pyridylamino)ethyl]thio}, and acetic acid) were positively correlated with G3–G2, and the major gut microbiota (*Ralstonia*, *Prevotella*, *UCG-005*, *Lactobacillus*, and *Prevotellaceae_NK3B31_group*) and the major metabolites (styrene, acetophenone, phenethylamine, citric acid, 5α-pregnan-3,20-dione, phosphoethanolamine, and maleic acid) were positively correlated with G6–G2. Additionally, we found that major gut microbiota (*Lactobacillus*, *Alloprevotella*, *Prevotella_9*, and *UCG-005*) and major metabolites (5α-Pregnan-3,20-dione, phosphoethanolamine, maleic acid, malic acid, GNK, 1,2,3-benzenetricarboxylic acid, GNK, and phosphoethanolamine) were negatively correlated in G6–G2 (Figures 6C2,C3).

## 4 Discussion

Good sleep contributes to physical and mental health, regulating emotions and metabolic function. Insomnia is a significant health issue in modern society (Yeung et al., 2022). Banxia–Shumi decoction, a classic Chinese herbal medicine with medicinal and edible homology used to treat insomnia for thousands of years—according to “Yin-Yang” and “Five Elements” theories that are the basis theories of TCM—guides Yang into Yin, clears middle energizers, and communicates Yin and Yang (Wang X. et al., 2024). In particular, adding Banxia–Shumi decoction to the conventional drug Eszolam is more effective at treating primary insomnia (Xuemin, 2020). Experiments have confirmed that Banxia–Shumi decoction can improve sleep structure by promoting the estrogen signaling pathway (Wu et al., 2022) and prolonging rapid eye movement sleep in rats (Wang Chenghao et al., 2023). It is still widely used in clinical practice and is safe and effective (Zhang et al., 2023).

Previous research by our group confirmed that Banxia-Yiyiren has specific sedative and hypnotic effects (Wang Liang et al., 2022), but the specific mechanism involved is still unclear. Modern pharmacological studies have shown that Banxia and Pinellia palmata have significant sedative and hypnotic effects (Lin et al., 2019; Guo et al., 2020). Research has shown that Banxia-Yiyiren has good anti-insomnia effects by regulating important neurotransmitters and sleep clock genes, while its metabolites, amino acids, and nucleotides may be essential materials for its anti-insomnia pharmacological effects (Yufeng Liu et al., 2023). The main components of Yiyiren can increase the number of sleeping animals, reduce their sleep latency, and increase their total sleep duration (Xiaoli et al., 2021); Yiyiren has pharmacological effects on promoting sleep quality and enhancing body immunity (Liang, 2019); and Yiyiren prepared via the traditional decoction method can exert sedative and sedative effects, the effects of which are positively correlated with its dosage (Haiyan, 2016). Chromatography–mass spectrometry analysis technology is fast, efficient, highly sensitive, and selective (Mingxi et al., 2021), providing strong technical support and high-quality data support for overcoming limitations in the study of the material basis and mechanism of action of TCMs.

We obtained qualitative data for Banxia-Yiyiren via UPLC-Q Exactive Orbitrap MS in both positive and negative ion detection modes and identified the main compound components, including amino acids, small peptides, nucleotides, organic acids, flavonoids, fatty acids, and lipids, via mass spectrometry. The EPM test has been applied to verify the antianxiety properties of Chinese herbal medicine (Murphy et al., 2010). The results of the EPM test revealed that Banxia-Yiyiren may increase people’s willingness to explore by improving anxiety-like symptoms caused by insomnia.

Modern pharmacological research has confirmed that Banxia is rich in active ingredients, such as alkaloids and flavonoids, which can have neuroprotective, sedative, and hypnotic effects on insomnia (Bai et al., 2022). Research has shown that the Banxia ethanol extract has sedative, hypnotic, and anticonvulsant pharmacological effects (Wu et al., 2011) and promotes sleep by increasing the number of REM sleep events, the number of transitions from NREM sleep to REM sleep, and the number of transitions from REM sleep to wakefulness (Lin et al., 2019). Yiyiren contains chemical components such as flavonoids, sugars, esters, unsaturated fatty acids, and lactams (Yang et al., 2022). Research has shown that amino acids are associated with GABAergic sleep/wakefulness regulation and that the ability of pallidin to regulate sleep in the surface glial cells of Drosophila depends on the availability of amino acids (Li X. et al, 2023). Another study revealed that flavonoids can significantly enhance the hypnotic effect of pentobarbital-induced sleep latency, and shortening and prolonging sleep times (Wang et al., 2012). Alkaloids have various pharmacological effects on improving sleep and combating depression, anxiety, and convulsions. Most flavonoids are quercetin and its derivatives (Kostikova et al., 2023). Flavonoids are important chemical components of plant-based sedative Chinese medicines that have pharmacological effects, such as improving sleep, combating depression and anxiety, and improving learning and memory (Peach et al., 2020).

In recent years, the gut microbiota has been shown to be an essential regulatory factor of the gut–brain axis that can affect brain function through neural, immune, and endocrine pathways, forming the microbiota–gut–brain axis (Lo et al., 2021). The gut microbiota is one of the hotspots in the study of neurological diseases and plays a role in the pathogenesis of insomnia (Zhang et al., 2021; Wang et al., 2022), and the relationship between insomnia and the gut microbiota has been elucidated (Feng et al., 2023). To date, relatively few studies have evaluated the relationships between gut microbiota and metabolism and insomnia (Han et al., 2022; Qi et al., 2022).

16S rRNA gene sequencing revealed significant differences in the abundance and diversity of the G2 microbiota compared with those of the G1 microbiota, indicating changes in the G2 microbiota. At the phylum level, compared with those in G1, the relative abundances of *Bacteroidota* and *Proteobacteria* in G2 increased, whereas the relative abundance of *Firmicutes* decreased. Recently, Mendelian randomization analysis of insomnia-related diseases revealed that a significant correlation between the class *Negativicutes*, *the* genus *Clostridium innocuum*, the genus *Dorea*, the genus *Lachnoclostridium*, the genus *Prevotella7*, and the order *Selenomonadales is associated* with an increased risk of insomnia (Li X. et al., 2023). Research has shown that *Bacteroidota*, *Proteobacteria*, and *Firmicutes* are associated with insomnia (Rizzatti et al., 2017; Wang et al., 2020; Agrawal et al., 2021; Zhang et al., 2022), and *Bacteroidota* and *Firmicutes* exhibit diurnal periodic changes (Liang et al., 2015). Therefore, *Bacteroidota* and *Firmicutes* are considered to be associated with improving sleep cycles to some extent. These findings need further verification via subsequent experiments. *Proteobacteria* are major pathogenic bacteria that primarily affect normal sleep by promoting inflammation and other pathways.

At the genus level, compared with those in G1, the relative abundances of *Prevotella_9*, *Prevotella*, *Ralstonia*, *Escherichia–Shigella*, and *UCG-005* in G2 increased, whereas the relative abundances of *Lactobacillus*, *Ligilactobacillus, Alloprevotella*, *Blautia*, *and Prevotellaceae_NK3B31_group* decreased. Research confirms that *Prevotella*, *Prevotella, UCG-005*, *Prevotellaceae_NK3B31_Group* and *Alloprevotella* can produce short-chain fatty acids (Scheperjans et al., 2015; Li et al., 2019), which are associated with the sleep circadian cycle and neuroinflammation (Fung et al., 2017; Tahara et al., 2018); *Escherichia*-*Shigella* has been shown to produce norepinephrine (Zhong et al., 2019), which regulates rapid eye movement sleep through complex mechanisms such as multiple pathways and factors (Mehta et al., 2016); and *Lactobacillus*, *Ligilactobacillus*, *Blautia*, and others are recognized probiotics that can effectively improve the gut microbial environment and reduce inflammation (Sovijit et al., 2021).

Fecal microbiota transplantation may be a novel treatment option for insomnia and may provide an alternative to traditional Chinese medicine (TCM) for treating this disease (Fang et al., 2023). These effects were positively correlated with increases in probiotic agents, such as Bifidobacterium, Lactobacillus, Turicibacter, and Fusobacterium. Compared with G2, treatment with Banxia-Yiyiren effectively increased the abundance of beneficial bacteria (*Firmicutes*, *Lactobacillus*, *Blautia*, etc.) and reduced the abundance of pathogenic bacteria (*Bacteroidota*, *Proteobacteria*, *Prevotella_9*, etc.) in insomnia model rats, thereby affecting neuroinflammation and neurotransmission and ultimately regulating sleep in insomnia model rats.

Fecal metabolites are considered products of cometabolism between the gut microbiota and the host. They not only reflect the state of the gut microbiota but also serve as a bridge between symbiotic bacteria and the host. Research has shown that the gut microbiota and fecal metabolites can strongly affect host health and the central nervous system (Zhang et al., 2020). Metabolomic analysis of the gut microbiota revealed good stability and reproducibility of the QC sample data via PCA. Moreover, the positive control drugs and Banxia-Yiyiren had no significant effect on the changes in the fecal metabolic profile, and 1,574 metabolites were identified and matched with the database. Research has revealed that children who go to bed early have significantly greater diversity and richness of the gut microbiota, as well as metabolic pathways, whereas gut microbiota metabolites are significantly reduced, which may be related to differences in the gut microbiota (Xiang et al., 2023). Our research results also reflect, to some extent, the significant differences in metabolites between PCPA-induced insomnia rats and normal rats. These findings provide a reference for our future plans and further research on the potential metabolic mechanisms of Banxia-Yiyiren in treating insomnia. PLS-DA does not result in “overfitting” and can better describe the sample, which can be used to search for model biomarker groups. The volcanic map revealed that there were significantly more differentially abundant metabolites between G1–G2 than between G3–G2 and G6–G2. The match rod graph revealed that there was mainly upregulation of cyclic acid and succinic acid between G1–G2 and downregulation of L-lysine and 8,8′-dicarboxy-1,1′-binaphthalene. G3–G2 mainly showed the upregulation of rhamnetin and 4-hydroxynordiazepam and the downregulation of succinic acid and erythrono-1,4-lactone, whereas G6-G2 mainly showed the upregulation of H-Trp-NH2 and stearic acid and the downregulation of succinic acid and erythrono-1,4-lactone. A correlation analysis and chord plot of the differentially abundant metabolites revealed that the correlation between the top 20 differentially abundant metabolites in G1–G2 was greater than that between G3–G2 and G6–G2. KEGG enrichment analysis of the top 20 differentially abundant metabolites in each group revealed that the main differentially abundant metabolites in each group were significantly enriched in various amino acid metabolism, ABC transport, protein digestion, and absorption pathways. For a long time, neurotransmitter imbalances, including imbalances in GABA, serotonin, dopamine, and norepinephrine, were believed to be the cause of insomnia. Research has shown (Gonulalan et al., 2020) that BDNF plays an important role in the function of the central nervous system. The primary metabolites of the Krebs cycle (citric acid, fumaric acid, succinic acid, pyruvate, malic acid, and citric acid analogs) are positively correlated with brain-derived neurotrophic factor (BDNF) activity, whereas the secondary metabolites associated with high expression of BDNF are flavonoids, flavonoids, naphthalenes, terpenoids, etc. Our study revealed primary metabolites such as citric acid, fumaric acid, and succinic acid in the mass spectrometry analysis of Banxia–Yiyiren, which is consistent with the results of previous reports. L-lysine is an essential amino acid in the body (Hayamizu et al., 2019) that can promote human development, enhance immunity, and improve anxiety and central nervous system function (Lei et al., 2023). Plasma copper-binding peptides containing L-lysine have multiple biological effects, including angiogenesis and nerve growth, and have antioxidant, anti-inflammatory, anti-pain, and anti-anxiety effects (Pickart et al., 2017). Research has shown that L-lysine has a similar effect as a serotonin receptor 4 antagonist and can effectively reduce anxiety and basal cortisol levels in rats (Jalal et al., 2022).

Correlation analysis between the fecal 16S rRNA microbiota and metabolomics revealed significant Pearson correlations (*p* < 0.05; *p* < 0.05; *p* < 0.05) between G1–G2, G2–G3, and G2–G6 at the genus level. After screening for |*ρ*| ≥ 0.8 and *p* < 0.05, we detected positive correlations between the differential microbial communities (*Blautia*, *UCG-005*, *Alloprevotella*, *Prevotella*, *Ralstonia*, and *Lactobacillus*) and the differentially abundant metabolites (rhamnetin, 4-hydroxynordiazepam, morin, melatonin, luteolin, styrene, acetophenone, phenethylamine, citric acid, and photosynthethanolamine). There was a negative correlation between *UCG-005* and differentially abundant metabolites (phosphoethanolamine, malic acid, malic acid, GNK, phosphoethanolamine, and 1,2,3-benzenetricarboxylic acid).

The metabolites produced by the gut microbiota can affect the metabolism and function of the CNS through neural, endocrine, and immune pathways. Research has shown that there is an increase in common *Bacteroides* and a decrease in *Firmicutes* in people with insomnia, and blue–green algae and rumen cocci belonging to the *Firmicutes phylum* are positively correlated with sleep quality (Xie et al., 2023). Research has shown that gut metabolites mediate the relationship between the microbiota and insomnia and can promote an important source of sleep signals (Han et al., 2022). Melatonin is a multifunctional neuroendocrine molecule secreted by the pineal gland that plays an important role in regulating circadian rhythms and sleep–wake cycles (Jia et al., 2023). It has antioxidant, neuroprotective, and immunomodulatory effects. Melatonin can prevent related diseases by regulating the host gut microbiota (Xu et al., 2017; Li X. et al, 2023).

Our previous bioinformatics research explored the network pharmacology and molecular mechanism of Banxia-Yiyiren in treating insomnia (Wang Q. et al., 2022). Several experimental studies have preliminarily confirmed that Banxia-Yiyiren may improve sleep by regulating the expression of serum inflammatory factors and the hippocampal neurotransmitters orexin and 5-HT and their receptors (Wang Liang et al., 2022); however, the specific pathway by which Banxia-Yiyiren regulates the gut microbiota and its metabolites in insomnia models is still unclear. As a traditional formula with the characteristic “medicinal food homology,” Banxia-Yiyiren can regulate sleep by affecting the composition, relative abundance, diversity, and metabolites of the relevant gut microbiota in PCPA-induced insomnia model rats. UPLC-Q Exactive Orbitrap MS analysis and metabolic analysis confirmed that Banxia-Yiyiren has an important effect on gut metabolites, especially citric acid and phenylalanine.

## 5 Conclusion

In summary, this study preliminarily verified that the PCPA-induced insomnia model is closely related to gut microbial metabolism and microecological disorders, and for the first time, we confirmed that Banxia-Yiyiren can act on the gut microbiota of PCPA-induced insomnia model rats, improving sleep and anxiety by regulating the species, structure, abundance, and metabolites of the gut microbiota. These findings provide a certain amount of gut microbiota and metabolic data for Banxia-Yiyiren to treat insomnia, which is highly important for further studies of the metabolic mechanism of Banxia-Yiyiren in the treatment of insomnia.

## Data Availability

The original contributions presented in the study are publicly available. This data can be found here: https://www.ncbi.nlm.nih.gov/, PRJNA1158529. For detailed testing procedures, please consult the first author Dr. Liang Wang and corresponding author Dr. Shaodan Li.

## Footnotes

^1^ https://www.genome.jp/kegg/pathway.html

^2^ https://hmdb.ca/metabolites

^3^ https://www.lipidmaps.org/

^4^ https://magic-plus.novogene.com/#/login
