# Supplementary figures and images for: Banxia-Yiyiren alleviates insomnia and anxiety by regulating the gut microbiota and metabolites of PCPA-induced insomnia model rats
Source: Front Microbiol. 2024 Nov 7;15:1405566. doi: 10.3389/fmicb.2024.1405566 (PMC11578828; doi:10.3389/fmicb.2024.1405566)

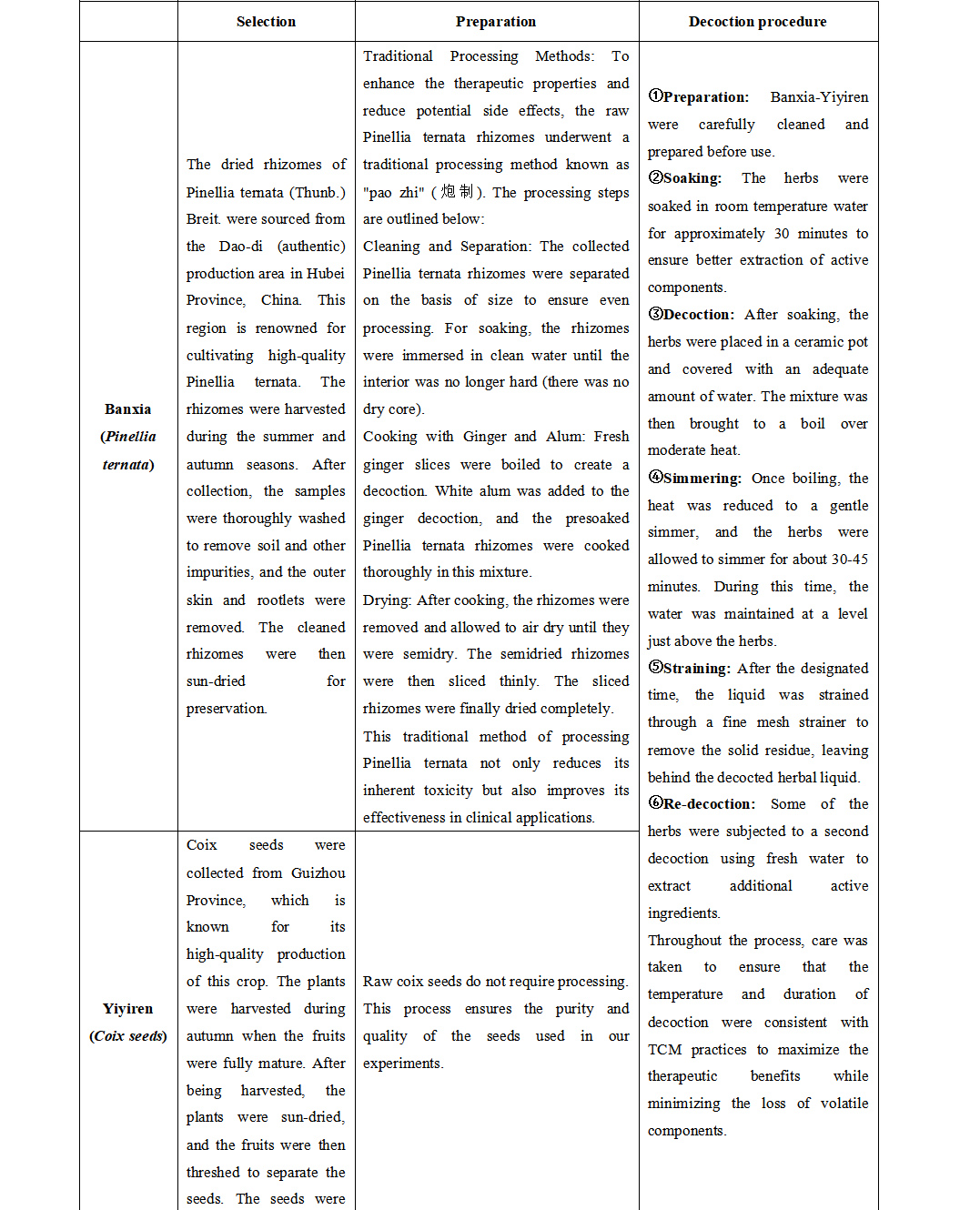

Supplement: Supplementary file 1 [file Data_Sheet_1.zip › Appendix 1-5/Appendix 1.jpg]

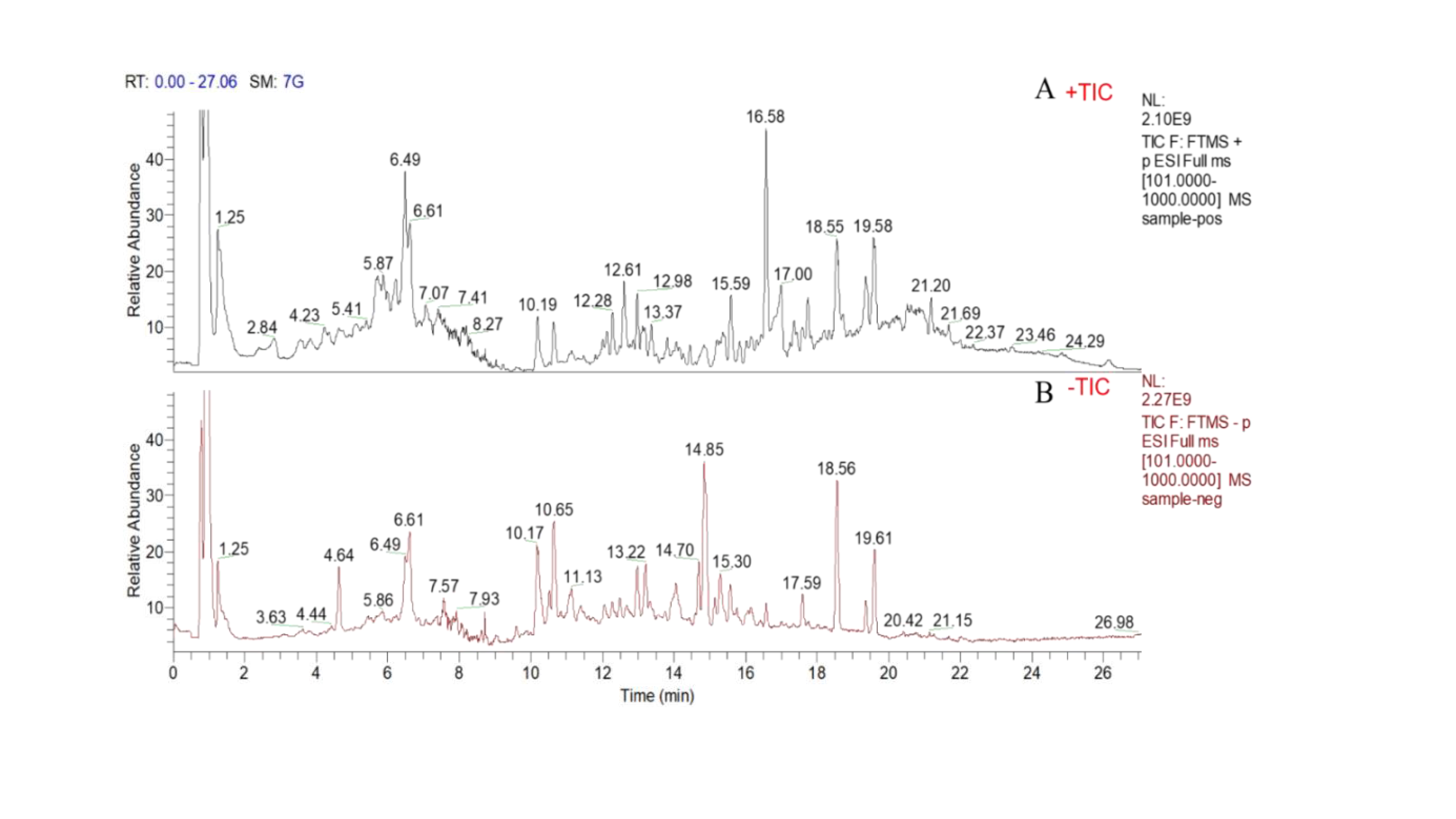

Supplement: Supplementary file 1 [file Data_Sheet_1.zip › Appendix 1-5/Appendix 2.jpg]

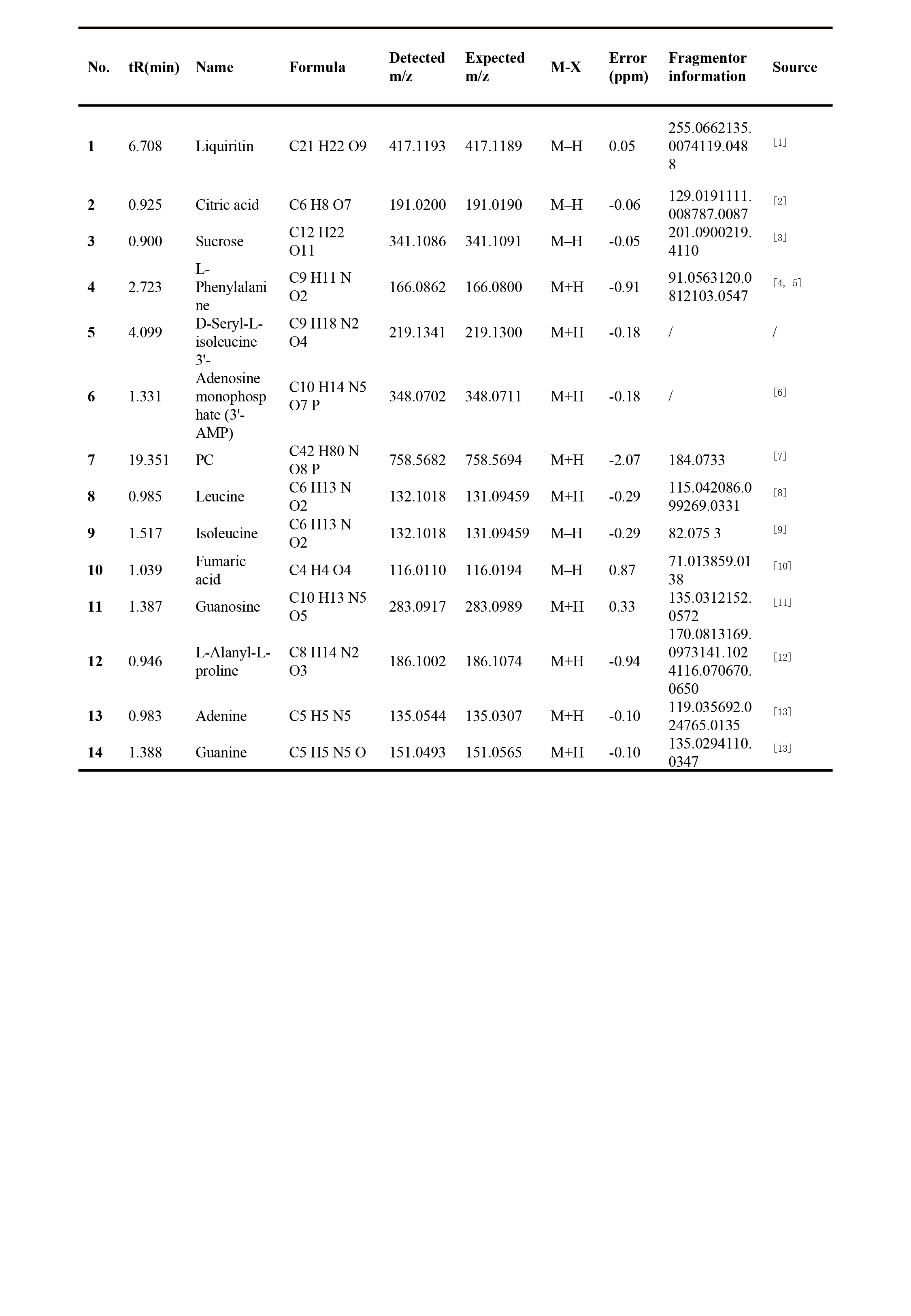

Supplement: Supplementary file 1 [file Data_Sheet_1.zip › Appendix 1-5/Appendix 3.jpg]

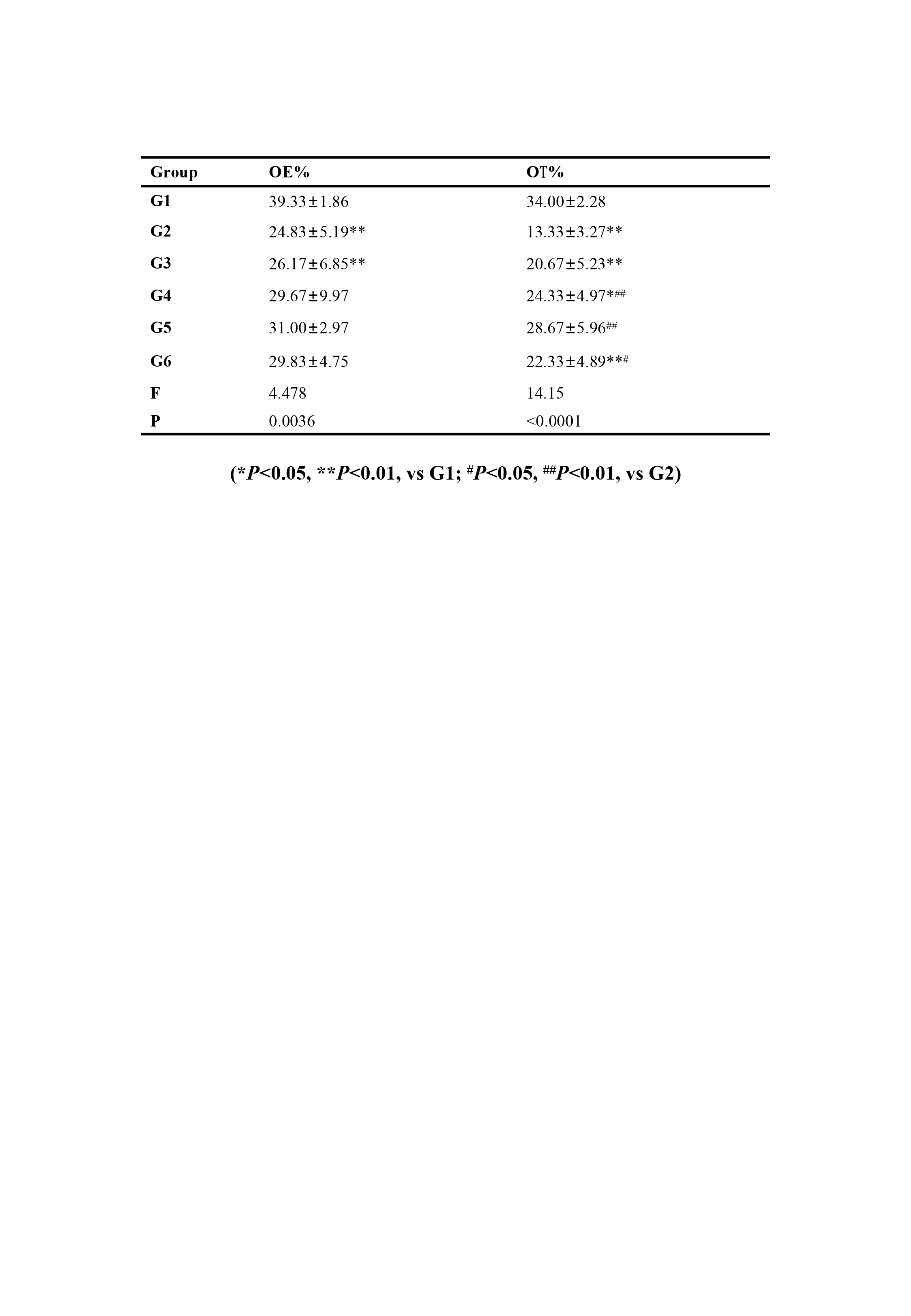

Supplement: Supplementary file 1 [file Data_Sheet_1.zip › Appendix 1-5/Appendix 4.jpg]

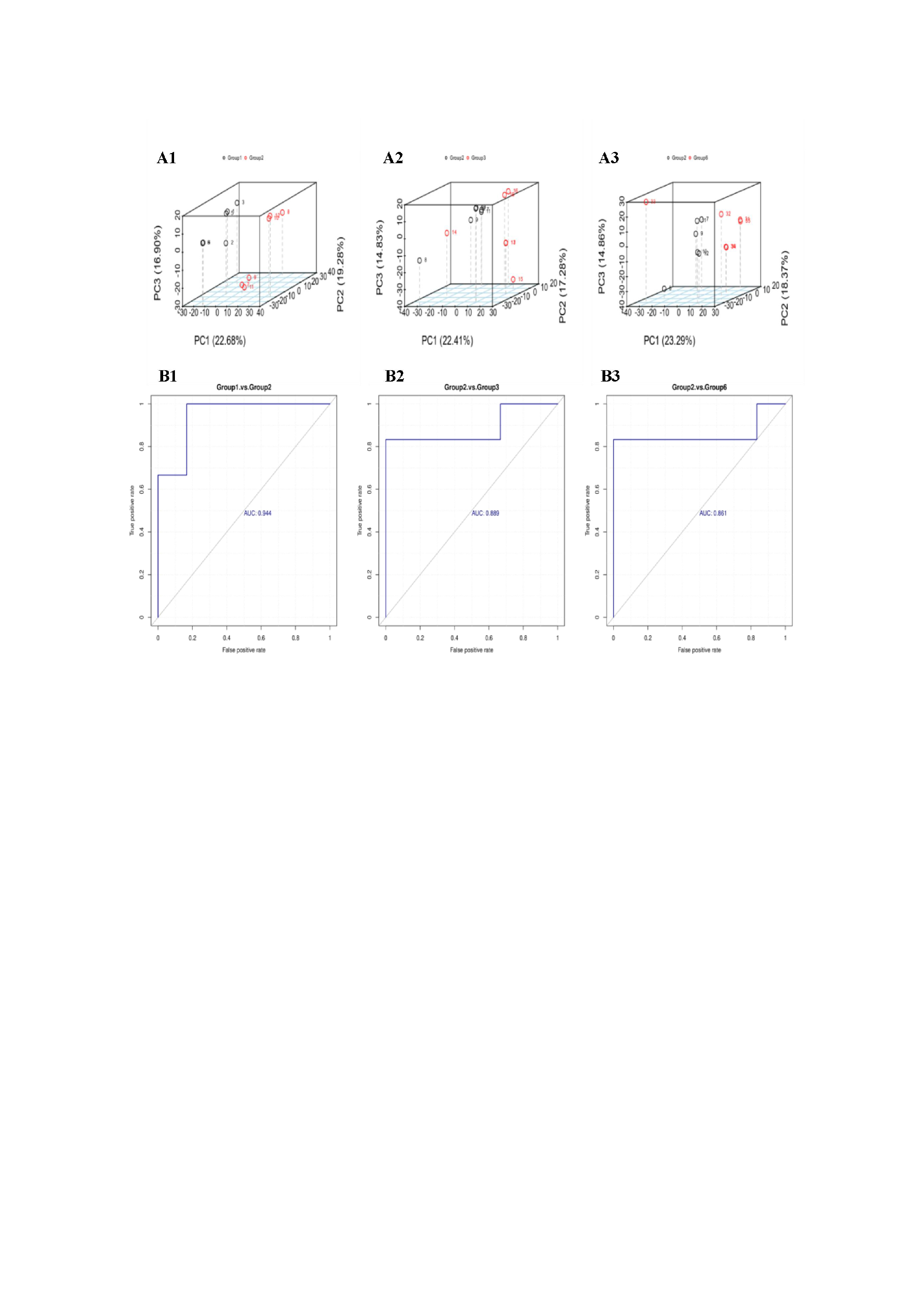

Supplement: Supplementary file 1 [file Data_Sheet_1.zip › Appendix 1-5/Appendix 5.jpg]
